# The association between cumulative adverse childhood experiences and ultra-processed food addiction is moderated by substance use disorder history among adults seeking outpatient nutrition counseling

**DOI:** 10.3389/fpsyt.2025.1543923

**Published:** 2025-03-27

**Authors:** David A. Wiss, Celine D. Tran, Erica M. LaFata

**Affiliations:** ^1^ University of Los Angeles California, Fielding School of Public Health, Department of Community Health Sciences, University of California, Los Angeles, Los Angeles, CA, United States; ^2^ Nutrition in Recovery LLC, Los Angeles, CA, United States; ^3^ Leonard Davis School of Gerontology, University of Southern California, Los Angeles, CA, United States; ^4^ Center for Weight Eating and Lifestyle Science, Drexel University, Philadelphia, PA, United States

**Keywords:** adverse childhood experiences, ultra-processed food, food addiction, eating disorder, substance use disorder, weight suppression, moderation

## Abstract

Adverse childhood experiences (ACEs), such as childhood maltreatment and household dysfunction, are positively linked to substance use disorders (SUD), weight loss efforts, and maladaptive eating behaviors, including ultra-processed food addiction (UPFA) and eating disorder (ED) symptoms. However, the differential association of ACEs with UPFA by lifetime SUD history and ACEs with EDs by weight suppression— the discrepancy between an individual’s highest and current weight/BMI in adulthood— have not been examined. Using logistic regression and marginal effects analysis, this cross-sectional study aimed to assess (1) cumulative ACEs as a risk factor for screening positive for UPFA and EDs, (2) lifetime SUD history as a moderator of the ACE-UPFA relationship, and (3) weight suppression as a moderator of the ACE-ED relationship. Among 287 adults presenting to a private practice offering nutrition counseling for EDs and SUD recovery, the presence of 4 or more ACEs (compared to <4 ACEs) significantly increased the odds of UPFA-positive screens (OR=1.99; CI=1.19-3.35; p=0.01) but not ED-positive screens (OR=1.36; CI=0.80-2.30, p=0.25). Additionally, the interaction between ACEs and SUD was significant to the UPFA outcome (p<0.01). Those with a self-reported lifetime history of SUD exhibited an increased probability of UPFA-positive screens in the presence of 4 or more ACEs. Meanwhile, the probability of UPFA-positive screens remained unchanged among those who did not report a lifetime SUD history. Cumulative ACEs did not significantly predict ED-positive screens, and the ACE-weight suppression interaction did not meet the threshold for significance. Overall findings underscore the cross-vulnerability between addictive behaviors and the potential importance of integrating nutrition interventions in addiction treatment for those with ACEs.

## 1 Introduction

Household adverse childhood experiences (ACE), measured by the highly utilized ACE scale, encompass childhood maltreatment and household dysfunction (1). Extensive research links higher ACE exposure to a wide range of psychosomatic consequences, including increased social disadvantage (2, 3), heightened risk of adverse health conditions (4–6), and greater likelihood of engaging in high-risk behaviors (1, 7, 8). With an estimated national economic burden of $13.9 trillion in lost healthy life-years (9), there is an urgent need for targeted interventions to mitigate the long-term health impacts of ACEs.

ACEs have also been associated with poor nutritional outcomes like higher body mass index (BMI) (10) and poor diet quality (11–13). As such, there is increasing concern regarding the link between ACEs and maladaptive eating behaviors, including ultra-processed food addiction (UPFA) and eating disorders (EDs). The Yale Food Addiction Scale defines UPFA as the compulsive consumption of highly palatable, ultra-processed foods [e.g., soft drinks, packaged sweet or savory snacks (14)] and the inability to reduce their intake in the face of negative consequences (15). Alternatively, ED behaviors encompass a wider range of harmful eating patterns, from compulsive overconsumption (i.e., bingeing) to severe restriction (16). While UPFA is biologically reinforced by the addictive potential of ultra-processed foods, EDs often involve distorted perceptions of food, weight, or appearance that drive disordered eating.

Theories suggest that ACEs contribute to UPFA and ED risk through neurobiological disruptions (17), such as executive dysfunction (18), stress dysregulation (19, 20), and altered reward processing (21). These vulnerabilities may heighten stress perception (22, 23), neurotic tendencies (24), and psychological distress (25) that, altogether, may promote reliance on maladaptive coping strategies such as ultra-processed food consumption. These foods may serve as one avenue of self-regulation due to their immediate, highly palatable, and rewarding nature (26–29). Ultra-processed foods may briefly alleviate distress by activating dopamine-driven reward pathways; however, the “relief” derived from these foods is short-lived, lending to reinforcement of their habitual use and overconsumption (30). Consequently, higher ACE scores have been linked to increased intake of ultra-processed foods high in calories, fat, and sugar (31, 32). Not only may overconsumption of these foods undermine an individual’s ability to maintain healthy eating habits (33), but it may also increase susceptibility to UPFA (34) and EDs (32). Bingeing on hyper-palatable, ultra-processed foods (35, 36), paired with efforts to restrain their eating, are common features of EDs posited to be sustained by the increasing availability of ultra-processed foods in the environment (37). Meanwhile, repeated ultra-processed food consumption may also become biologically reinforced and progress toward addiction (15).

Research has linked higher ACE scores to increased symptoms of UPFA (32, 38, 39) and EDs (40–44), as well as ED diagnoses (45, 46). Treatment-seeking individuals with EDs also reported higher ACE exposures compared to a nationally representative sample (47). However, studies have yet to assess whether ACE exposure predicts screening outcomes using instruments designed to identify UPFA and ED risks at predetermined cut-points. Such tools are essential for efficiently identifying patients who need further evaluation and treatment to mitigate health risks. Additionally, while ACEs have been independently associated with both UPFA and EDs, these relationships have not been examined within the same study population, limiting direct comparisons of ACE-related risk across eating pathologies.

There is also a need to explore moderators in the ACE, UPFA, and ED relationships. While research on moderating ED symptoms is limited (41, 42, 44), no studies have examined moderators of UPFA. A common limitation is the frequent use of ACEs as a continuous variable, which makes it difficult to identify dichotomous interacting factors in these relationships. This is particularly relevant for clinicians who rely on screening instruments with predetermined cut-points to assess risk and guide clinical decision-making. This approach also overlooks the significant impact of cumulative ACEs (22, 24), where prior research suggests that dichotomizing ACEs at a threshold of four or more exposures better predicts adverse outcomes than assessing individual ACEs (4, 6). While some studies have applied a dichotomized model to assess the ACE-ED relationship (40, 45, 48), this approach has not been applied to the ACE-UPFA relationship.

One potential moderator of the ACE-UPFA relationship is substance-use disorder (SUD). While ACEs independently increase the risk of both SUDs (49, 50) and UPFA, the intersection of these constructs remain unexplored. Individuals with a history of SUD may be particularly vulnerable to UPFA (51) as ultra-processed foods possess addictive qualities by activation of the dopamine system (or reward pathways) that mirror those engaged by drugs (52). Over time, frequent drug use and ultra-processed food consumption may lead to shared disruptions in dopamine signaling and enhance general reward-seeking behavior (53). Shared consequences may entail desensitization to natural, non-substance-related rewards (e.g., physical activity, hobbies, social interactions) and weakened inhibitory control over the consumption of highly reinforcing stimuli that promote intense feelings of pleasure [i.e., drugs (54) and ultra-processed foods (55, 56)]. While research has linked substance use to UPFA symptoms (57), the potential for cross-vulnerability between SUD and UPFA remains unclear (58, 59).

It may be the case that individual factors, such as ACE exposure, may contribute to the co-occurrence or transition between SUD and UPFA. Blunted reward processing (60) and impulsivity in response to negative affect (61) have mediated direct links between early life adversities and problematic substance use. Early life adversities have also shown a potential to amplify drug-related cravings (62, 63) and neural sensitivity to psychostimulants (23). Meanwhile, similar adaptations in reward-related neurocircuitry (64) and dysregulations of affect (65) are implicated in associations of early life adversities with UPFA and hyperpalatable food cravings (66). In this context, ACE-related disturbances may heighten susceptibilities to compulsive consumption patterns targeting various highly rewarding stimuli. In combination, exposure to ACEs and having a lifetime history of SUD may compound neurological and behavioral vulnerabilities that lend to UPFA risk.

Weight suppression has yet to be examined as a moderator in the ACE-ED relationship. While ACEs are linked to greater ED symptoms, weight suppression—the discrepancy between an individual’s highest and current weight/BMI after reaching adulthood (67)— is associated with ED diagnoses (68), symptom maintenance (69, 70), and clinical impairment (71). Weight suppression is often considered an adaptive subconstruct of dieting that entails engaging in restraint, restriction, or compensatory behaviors to both achieve weight loss and counteract psychobiological pressures to regain weight (72). Evidently, weight suppression has been linked to greater weight gain (73, 74), increased metabolic efficiency or reduced caloric needs (73), hormonal appetite dysregulation (75–77), and heightened reinforcing value of food (76). Unwanted weight gain—or even its perceived risk—may conflict with ED-related goals, such as maintaining a lower weight and conforming to internalized beauty standards. In vulnerable individuals, weight suppression—initially achieved through adaptive dieting (e.g., eating low-fat foods)—may trigger maladaptive dieting (e.g., purging) and heighten ED risk (78). Accordingly, greater weight suppression has been linked to more severe ED psychopathology, including poorer self-esteem related to weight and appearance (79–82).

Examining weight suppression alongside individual factors may help identify at-risk groups for EDs following significant weight loss. Specifically, ACE exposure may not only promote weight-suppressive behaviors but also heighten vulnerabilities to ED symptoms in weight-suppressed individuals. ACEs are linked to higher BMIs (10), which may promote chronic dieting and weight cycling (83). Additionally, ACEs and weight suppression share associations with increased concerns about eating, weight, and body shape (44), as well as low-self-esteem. The latter of which has been shown to mediate links between ACEs and binge-eating (84). Additionally, weight suppression is believed to reduce satisfaction related to food intake (76, 85), mirroring blunted reward sensitivity observed in ACEs. This effect is particularly relevant in cases where those with binge-type EDs exhibit satiation deficits compared to those without EDs (86). Women with a history of anorexia nervosa and bulimia nervosa also demonstrate difficulty distinguishing the emotional value between positive and negative feedback in monetary reward tasks (87, 88). Therefore, in the context of ACEs, individuals with weight suppression may be particularly susceptible to EDs.

Taken together, this study aims to address key gaps in the literature by assessing how cumulative ACE exposure relates to UPFA and ED screening outcomes, along with potential moderators in these relationships. First, no research has assessed cumulative ACEs (dichotomized at 4 or more ACEs) in relation to UFPA and ED risk within the same sample, which would allow for direct comparisons of ACE-related risk across eating pathologies. Second, several moderators of cumulative ACE-related risk remain unexplored, and assessing dichotomized moderators in these relationships may improve the detection of these effects. Specifically, the presence of lifetime SUD history may amplify ACE-related vulnerabilities to UPFA, which may reveal cross-vulnerability potential across addictive behaviors. Additionally, being weight suppressed may heighten ACE-related ED risk, highlighting high-risk subgroups that may be particularly susceptible to disordered eating. We hypothesized that 1) individuals with 4 or more cumulative ACEs are more likely to screen positive for both UPFA and ED risk; 2) self-reported lifetime SUD history would strengthen the relationship between cumulative ACEs and UPFA-positive screens; and 3) being weight suppressed would also strengthen the relationship between cumulative ACEs and ED-positive screens.

## 2 Methods

This study was approved by the UCLA Institutional Review Board (IRB# 20-008829) to collect data from September 2020 to April 2024. Data were sourced from a private, cash-based nutrition counseling practice in Los Angeles, California, where registered dietitian nutritionists specialize in EDs and SUDs. The patient population primarily sought nutrition support for disordered eating or SUD recovery.

Data were collected at a single time point via a HIPAA-compliant online intake form, completed independently before the initial consultation. All new patients received an email with a link to a questionnaire covering demographics, self-reported SUD status, and screening assessments for ACEs, UPFA, and EDs. Demographic data included age, gender, race/ethnicity, education, parental education, and self-reported height and weight (including highest and lowest adult weights) to calculate BMI and weight suppression.

The final analysis, conducted in April 2024, included 287 participants (73.9% women), ages 21–75. Participants were at least 21 years old to ensure accurate weight suppression reporting. Only those who provided written informed consent were included. A total of 20.7% of potential participants opted out. There were no missing data; however, two participants were excluded for reporting a highest lifetime weight lower than their lowest (implausible data assumed participant error).

Adverse childhood experiences: Participants completed the 10-item ACE questionnaire with yes/no responses (1). A threshold of 4 or more “yes” responses (4+ ACEs) categorized participants as having high cumulative ACEs, while those with fewer than 4 (<4 ACEs) were classified as having low cumulative ACEs. The 10-item ACE scale has demonstrated good internal consistency for use in adults (89).

Modified yale food addiction scale (mYFAS2.0): The mYFAS2.0 (90) is a validated 13-item shortened version of the original 35-item YFAS (15). Two items assess clinical significance, requiring at least one positive response for UPFA classification. Severity levels were defined as mild (2–3 symptoms), moderate (4–5 symptoms), or severe UPFA (6+ symptoms). Participants were dichotomized into two groups: none/mild UPFA and moderate/severe UPFA, with the latter indicating a UPFA-positive screen or having UFPA risk.

To enhance UPFA classification specificity, we adopted a higher dichotomization threshold for our sample presentation, which largely constitutes elevated baseline symptomatology. This aligns with studies using stricter criteria in populations [e.g., binge-eating disorder (91)] that may exhibit compulsive eating not necessarily rooted in addiction-like processes.

Eating disorder examination – questionnaire short (EDE-QS): The EDE-QS is a validated 12-item version (92) of the original 28-item EDE-Q (93). Participants rated the frequency of certain behaviors over the past 7 days on a 4-point scale: (1) 0 days; (2) 1–2 days; (3) 3–5 days; and (4) 6–7 days. Scores range from 0 to 36, with a threshold of 15 or higher indicating a high likelihood of an ED based on a sensitivity of 0.83 and specificity of 0.85 (94). Participants scoring above this threshold were categorized as an ED-positive screen or having ED risk.

Lifetime substance use disorder history: Participants were asked, “Do you identify as having a current or previous alcohol or other substance use disorder?” Responses were categorized as “yes” (self-reported lifetime history of SUD) or “no.”

Weight suppression: Weight suppression was calculated by dividing current weight by the lifetime adult midpoint weight (average between highest and lowest reported adult weights) (95). This calculation was preferred over the more common calculation (i.e., the absolute difference between highest and current adult weight), as the latter may be less sensitive in distinguishing ED from UPFA symptoms. This distinction is relevant given the associations between weight suppression and ultra-processed food intake (95) and the overlap between UPFA and ED characteristics (96).

Weight suppression was categorized as a binary: participants above the sample mean for weight suppression were considered weight suppressed, while those below the sample mean were categorized as not weight suppressed. Dichotomizing by the mean accounted for the sample’s skew toward individuals with a lifetime SUD history [linked to weight changes (97)] and higher BMIs.

### 2.1 Statistical analysis

Using Stata 18 (98), we conducted logistic regression analyses to evaluate two hypothesized main effects and two hypothesized interaction effects. Logistic regression models were estimated using Maximum Likelihood Estimation (p<0.05). Results were reported as odds ratios (ORs) and 95% confidence intervals (CIs).

All predictors (cumulative ACEs), moderators (lifetime SUD history, weight suppression status), and outcome variables (UFPA risk, ED risk) were binary (4+ ACEs vs. <4 ACEs). All models were also adjusted for potential confounders. Continuous covariates included age and BMI. Binary covariates included gender (female vs. non-female) and race (White vs. non-White). Due to small sample sizes, men and nonbinary participants were coded as non-female, and non-Hispanic Black, Hispanic/Latino, Asian, Other/Mixed, or those selecting “prefer not to say” were coded as non-White. Additional categorical covariates included education (high school or less, some college, college graduate, graduate-level) and parental education (at least one parent college graduate vs. none).

For Hypothesis 1, we assessed direct relationships using two separate logistic regression models: one examining the association between cumulative ACEs and UPFA-positive screens and the other between cumulative ACEs and ED-positive screens. For Hypothesis 2, we updated the ACE-UPFA model to include an interaction term between cumulative ACEs and a self-reported lifetime history of SUD. For Hypothesis 3, we updated the ACE-ED model to include weight suppression status as a moderator.

For Hypotheses 2 and 3, interaction terms were assessed within the logistic regression framework and further analyzed via joint marginal effects analysis. The significance of the latter was determined using post-estimation Wald tests (p<0.05). Predicted probabilities with 95% CIs were reported.

## 3 Results

**Table 1** reveals that the majority of our 287 participants were women (73.9%) and White (82.2%), with a mean age of 40.2 (SD=13.6). Over half (55.4%) had a BMI above 25 (4.9% underweight, 39.7% normal, 18.1% overweight, 37.3% 30 or above), with a mean BMI of 28.7 (SD=8.9).

**Table 1:** Demographic characteristics of study sample by adverse childhood experience exposure (N=287; Ages 21+).

Characteristic	N (%)	<4 ACEs n (%) (n=143)	4+ ACEs n (%) (n=144)	p-value
**Age (years)**				0.14
18-29	74 (25.8)	42 (29.4)	32 (22.2)	
30-39	86 (30.0)	37 (25.9)	49 (34.0)	
40-49	47 (16.4)	28 (19.6)	19 (13.2)	
50+	80 (27.9)	36 (25.2)	44 (30.6)	
**Gender**				0.21
Not Woman	75 (26.1)	42 (29.4)	33 (22.9)	
Woman	212 (73.9)	101 (70.6)	111 (77.1)	
**Race/Ethnicity**				0.29
Not White	51 (17.8)	22 (15.4)	29 (20.1)	
White	236 (82.2)	121 (84.6)	115 (79.9)	
**Education**				0.05
HS or Less	25 (8.7)	9 (6.3)	16 (11.1)	
Some College	73 (25.4)	29 (20.3)	44 (30.6)	
College	110 (38.3)	63 (44.1)	47 (32.6)	
Graduate School	79 (27.5)	42 (29.4)	37 (25.7)	
**Parental Education**				0.02*
Not College Grad	83 (28.9)	32 (22.4)	51 (35.4)	
College Grad	204 (71.1)	111 (77.6)	93 (64.6)	
**BMI**				0.49
Underweight	14 (4.9)	6 (4.2)	8 (5.6)	
Normal Weight	114 (39.7)	60 (42.0)	54 (37.5)	
Overweight	52 (18.1)	29 (20.3)	23 (16.0)	
Obesity	107 (37.3)	48 (33.6)	59 (41.0)	
**Lifetime SUD**				0.00**
No	124 (43.2)	80 (56.0)	44 (30.6)	
Yes	163 (56.8)	63 (44.1)	100 (69.4)	
**Weight Suppressed**				0.17
Below Average	151 (52.6)	81 (56.6)	70 (48.6)	
Above Average	136 (47.4)	62 (43.4)	74 (51.4)	

ACEs, Adverse Childhood Experiences; HS, High School; BMI, Body Mass Index; SUD, Substance Use Disorder.

*Significant chi-squared at p<0.05 comparing <4/4+ ACEs; **significant at p<0.01.

Half of the participants reported 4+ ACEs (50.2%), with this group significantly more likely to report a lifetime history of SUD (69.4% vs. 44.1% for those with <4 ACEs; p<0.01). Age, gender, race/ethnicity, education, and BMI did not correlate with ACE scores, but parental education did (p=0.02 with lower levels of education in the 4+ ACE group).

Concerning our outcomes of interest, 40.4% met the criteria for moderate or severe UPFA (50.2% none; 9.4% mild; 8.7% moderate; 31.7% severe), 61.7% had an ED-positive screen, and 36.2% met the criteria for both UPFA and ED risk. 56.8% of participants reported a lifetime history of SUD, while 47.4% reported above-average weight suppression.

**Hypothesis 1:** **Table 2** shows that participants with 4+ ACEs had significantly higher odds of UPFA-positive screens than those with <4 ACEs (OR=1.99; CI=1.19-3.35; p=0.01). However, cumulative ACEs did not significantly predict the odds of ED-positive screens (OR=1.36; CI=0.80-2.30; p=0.25).

**Table 2:** Adjusted logistic regression of adverse childhood experiences on positive screens for ultra-processed food addiction and eating disorder (N=287; Ages 21+).

4+ ACEs	OR	95% CI	p-value
Ultra-Processed Food Addiction	1.99	1.19 - 3.35	0.01*
Eating Disorder	1.36	0.80 - 2.30	0.25

ACEs, Adverse Childhood Experiences; OR, Odds Ratio; CI, Confidence Interval.

Models adjusted for: age, gender, race/ethnicity, education, parental education, body mass index.

*Significant at p<0.05.

**Hypothesis 2:** **Supplementary A** shows that interaction terms between 4+ ACEs and self-reported lifetime history of SUD (OR=2.50; 95% CI=0.84-7.49, p=0.10) were jointly significant (p<0.01) in the post-estimation analysis of their joint marginal effects.

**Figure 1:**
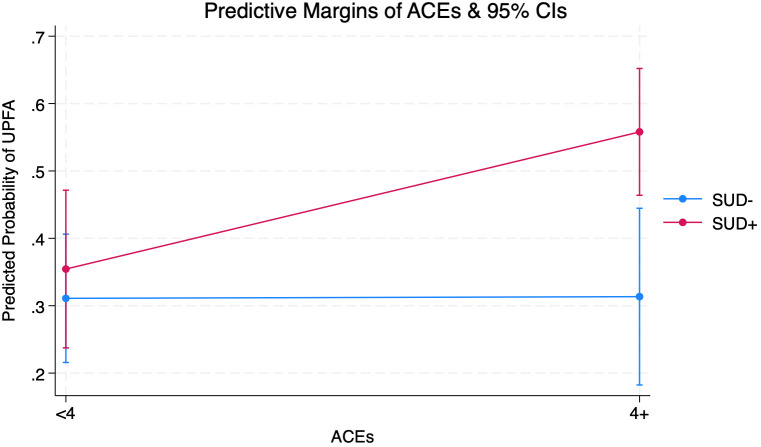
Margins Plot from Adjusted Logistic Regression Interacting Adverse Childhood Experiences (ACEs) and Substance Use Disorder (SUD) on Ultra-Processed Food Addiction (UPFA) Among Adults (Ages 21+) Seeking Nutrition Counseling (N=287) (see **Supplementary A** for full output).

**Figure 1** shows that among individuals who reported a lifetime SUD history, predicted probabilities of UPFA-positive screens were 35.4% (CI=0.23-0.47) for those with <4 ACEs and 55.8% (CI=0.46-0.65) for those with 4+ ACEs, representing a 20% increase across ACE scores. Individuals who did not report a lifetime history of SUD showed no change across ACE scores in UPFA-positive screens. Those with self-reported lifetime SUD history had greater predicted probabilities of UPFA-positive screens regardless of ACEs.

**Hypothesis 3:** **Supplementary B** shows that the interaction between weight suppression status and cumulative ACEs was non-significant (OR=1.70; CI=0.60-4.80; p=0.32). A post-estimation test of joint marginal effects also showed no significant interaction (p=0.18).

**Figure 2:**
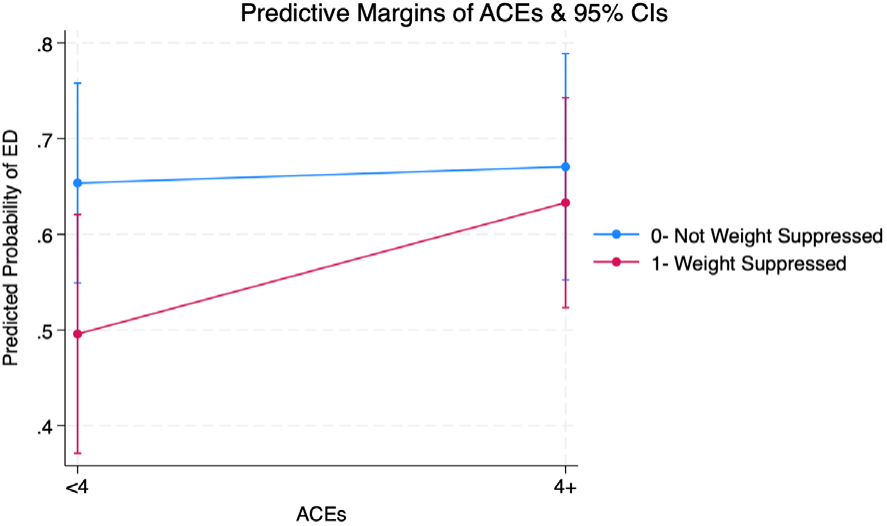
Margins Plot from Adjusted Logistic Regression Interacting Adverse Childhood Experiences (ACEs) and Weight Suppression on Eating Disorder (ED) Among Adults (Ages 21+) Seeking Nutrition Counseling (N=287) (see **Supplementary B** for full output).

**Figure 2** shows that among individuals with weight suppression, predicted probabilities of ED-positive screens were 49.6% (CI=0.37-0.62) for with <4 ACEs and 63.3% (CI=0.55-0.78) for those with 4+ ACEs, representing a 14% increase across ACE scores. Among individuals without weight suppression, the predicted probability of ED-positive screens showed no changes across ACE scores. Those without weight suppression also had greater predicted probabilities of ED-positive screens regardless of ACEs.

## 4 Discussion

### 4.1 Ultra-processed food addiction

In alignment with prior research (32, 38, 39), our findings suggest that 4+ ACEs are associated with a greater likelihood of meeting the criteria for UPFA. One possible explanation is that children exposed to adversity often lack access to effective coping strategies or positive models of self-regulation in early life, leaving them more vulnerable to developing maladaptive behaviors. As a result, ultra-processed foods—being highly accessible and immediately rewarding—may become a primary means of self-regulation in early life (30). ACE-related effects may reinforce this behavior through several pathways. One possibility is that these individuals with high ACE exposure turn to ultra-processed foods to manage distress, given the lasting impact of ACEs on stress and emotional regulation (23, 24). Another involves ACE-related reward dysfunction (21), where blunted pleasure responses to naturally rewarding activities may drive increased consumption of ultra-processed foods for their potent dopamine-releasing effects. Over time, repeated ultra-processed food exposure may cause chronic dopaminergic hyperactivation and subsequent downregulation of dopamine receptors involved in reward processing, ultimately reinforcing dependency through neurobiological reinforcement (53). As a result, individuals with high ACEs may rely on ultra-processed foods not only for self-regulation and pleasure (99) but also to maintain baseline reward function and avoid discomfort when intake is reduced.

Notably, the interaction between cumulative ACEs and a lifetime history of SUD more than doubled the odds of UPFA-positive screens. While prior studies have struggled to establish links between SUDs and UPFA (58, 59), our findings suggest that elevated ACE exposure may promote cross-vulnerabilities between SUD and UPFA. The enduring effects of ACEs combined with SUD-related dopaminergic dysregulation (53) may amplify disruptions in pleasure perception (60) beyond those seen in individuals with either risk factor alone. Cross-vulnerabilities between SUD and UPFA may be particularly relevant during early SUD recovery when withdrawal symptoms are most intense, prompting individuals to seek out ultra-processed foods as a substitute for diminished sources of gratification (100). The same dopaminergic impairments that contribute to substance addiction may also drive addictions to ultra-processed foods, reinforcing a new cycle of dependency that targets food instead of drugs. This effect may be particularly pronounced in individuals with ACE-related reward deficits, as the added neurobiological strain from substance use may further intensify drives for highly rewarding stimuli such as ultra-processed foods.

### 4.2 Eating disorders

Contrary to our hypothesis, cumulative ACEs did not increase the likelihood of ED-positive screens in our sample. One explanation may be the loss of predictive power from using logistic regression and dichotomized screening instruments. This contrasts with prior work linking ACEs to EDs, which assessed continuous ACE measures (42, 46, 47) and individual ACE indicators (45) as predictors or assessed ED symptom count-based severity (47) and specific ED symptoms as outcomes (40–43). Another possibility is that ACEs predicted UPFA but not EDs due to our sample composition, where 56.8% reported a lifetime history of SUD, and 55.4% had a BMI above 25. Lifetime SUD history may have compounded ACE-related reward dysfunction, increasing vulnerability to compulsive eating. Similarly, obesity has been linked to compulsive eating through shared reward-processing vulnerabilities with addiction (101). As a result, our sample presentation may have been skewed toward reward-driven, compulsive consumption patterns, better captured by the mYFAS, rather than other traditional ED features like pathological dieting, better assessed by the EDE-QS. Thus, the relevance of a general screening measure such as the EDE-QS to our sample may be limited.

The EDE-QS also broadly assesses disordered eating within the context of ED-specific psychopathology, including concerns about weight, shape, or appearance. Certain EDs (namely binge-type EDs) also feature compulsive overconsumption patterns relevant to our sample, albeit unspecific to ultra-processed food intake. In contrast, UPFA is characterized by compulsive eating of specifically ultra-processed foods, driven by neurobiological reinforcement and independent of ED-related distress or body image concerns (102). As a result, the EDE-QS may have also failed to detect cases of compulsive overconsumption (i.e., binging) that lacked guilt or ED-related concerns but were also not rooted in addiction-like processes. This may have led to an underestimation of ED risk in our sample and weakened the observed ACE-ED association, lending to our contrasting findings with other studies linking higher ACEs to binge eating (40, 43).

Certain unmeasured factors may have also differentially influenced ACE associations with ED and UPFA risk. Specifically, adulthood adversities like food insecurity, economic instability, or limited access to healthcare/mental health resources may be potential confounders in the ACE-ED relationship. Food insecurity, for example, has been shown to exacerbate the ACE-binge eating relationship in bariatric surgery-seeking patients (42). In contrast, our sample—composed of primarily socially advantaged White women with higher educational attainment and socioeconomic status—may face fewer related stressors, potentially weakening the ACE-ED link. Alternatively, ACE-related UPFA risk may be sustained by 1) the strength of biological reinforcement that entails addiction and 2) the modern food environment, where ultra-processed foods are widely available and heavily marketed, lending to the sustenance of cravings even in the absence of distress-related triggers (30). Many participants were also engaged in other forms of mental health treatment (e.g., therapy), which may have attenuated patterns of disordered eating related to ACEs upon presentation to nutrition counseling. Relatedly, greater self-compassion has been shown to weaken the ACE-ED relationship (41), while emotion regulation strategies may mitigate ACE-related psychological distress (25). Meanwhile, ED and SUD treatments are more established than those for UPFA, which remains an evolving construct and is less likely to be formally recognized in clinical care.

Given the lack of a significant main effect, it was unsurprising that the interaction between ACEs and weight suppression was non-significant in predicting ED risk. However, among weight-suppressed individuals, those with 4+ ACEs showed a trend toward a higher likelihood of ED-positive screens, suggesting a potential compounded risk. Interestingly, individuals without weight suppression consistently had higher predicted probabilities of ED-positive screens, regardless of ACEs. It may be the case that the prominence of addictive-like or compulsive consumption patterns, likely influenced by lifetime SUD history, may have overshadowed the predictive power of weight suppression for ED risk in our sample (where 49.8% met the criteria for mild to severe UPFA). Weight suppression has been previously linked to restrictive-type EDs, such as anorexia nervosa and bulimia nervosa, but not binge-eating disorder (68), which shares behavioral and neurological similarities with UPFA (102). Theories also suggest that UPFA represents intensified ED severity (96), and recent findings indicate that UPFA symptoms mediate 73.8% of the positive association between ultra-processed food intake and ED symptomology (32). Additionally, research suggests that weight suppression may be a stronger predictor of disordered eating in individuals with lower BMIs (<21.5 kg/m² (103);), a group underrepresented in our sample, where only 4.9% were underweight, and 55.4% had a BMI of 25 or higher. Efforts to suppress weight exist in our sample but are likely more difficult when addictions are present.

### 4.3 Limitations, future considerations, and conclusions

Limitations include the cross-sectional design, which restricts causal inference, as well as the use of logistic regression with screening instruments dichotomized at cut-points, which may reduce the nuance of dose-response associations among our scales (104). The 10-item ACE scale focuses on family and household dysfunctions and may overlook community (e.g., bullying/teasing in school) and systemic (e.g., food insecurity related) adversities that could be more relevant to ED risk in our sample. Additionally, the EDE-QS may under-detect binge-type EDs, while the mYFAS2.0 might overrepresent UPFA-positive screens due to behavioral overlaps with binge-type EDs (105). The mYFAS2.0 may also misclassify underweight individuals and those with restrictive EDs as UPFA-positive screens due to shared negative perceptions about food intake (106).

The sample consists of individuals seeking nutritional management for SUD/EDs, so findings may not be generalized to other ED populations. Additionally, the private nutrition counseling practice lacked access to medical records and on-site clinicians qualified to diagnose SUD or EDs. Consequently, reliance on retrospective screening tools and self-reported intake questionnaires introduces recall bias and limits findings to risk assessment rather than formal diagnoses. Furthermore, our sample primarily consists of women of higher-socioeconomic status, a common limitation in existing literature. While socioeconomic factors, such as food insecurity, may have influenced our findings, they were not explicitly measured in our study. Consequently, the generalizability of our results to lower-income or food-insecure populations remains limited.

Future longitudinal studies are needed to clarify causal links among ACEs, SUD, weight suppression, UPFA, and EDs. Research should incorporate comprehensive assessments, including clinician-administered interviews, of SUD and disordered eating. The latter may reduce bias toward restrictive EDs and better capture the impact of weight suppression on ED risk in those with childhood adversity. Examining additional moderators and mediators may further elucidate pathways between ACEs and maladaptive eating and identify vulnerability characteristics. Greater demographic diversity is also needed to improve generalizability across genders, socioeconomic backgrounds, and cultures. Beyond research, current nutritional interventions may benefit from incorporating ACE screenings to assess the risk for addictive-like eating patterns (107). Integrating nutritional counseling into addiction treatment could also help improve dietary habits and may prevent the progression of maladaptive eating behaviors to UPFA (108). It could also prove worthwhile to ascertain whether UPFA confers additional risk for SUD relapse.

In conclusion, our research underscores the importance of a comprehensive approach to understanding how the psychological impact of ACEs affects adult eating behavior and the role of SUD in perpetuating maladaptive consumption patterns. We found that individuals with a history of greater ACEs have an increased risk of screening positive for UPFA, particularly when these experiences co-occur with a self-reported lifetime history of SUD. Our findings suggest that SUD may exacerbate the risk of UPFA by amplifying the impact of childhood adversity and highlight the need for targeted interventions to address the interconnected issues of ACEs, SUD, and maladapted eating behaviors.

## Data Availability

The raw data supporting the conclusions of this article will be made available by the authors, without undue reservation.

## References

[1] Felitti VJ Anda RF Nordenberg D Williamson DF Spitz AM Edwards V. Relationship of childhood abuse and household dysfunction to many of the leading causes of death in adults the adverse childhood experiences (ACE) study. Am J Prev Med. (1998) 14:245–58. doi: 10.1016/s0749-3797(98)00017-8 9635069

[2] Metzler M Merrick MT Klevens J Ports KA Ford DC. Adverse childhood experiences and life opportunities: Shifting the narrative. Child Youth Serv Rev. (2017) 72:141–9. doi: 10.1016/j.childyouth.2016.10.021 PMC10642285 37961044

[3] Skiendzielewski K Forke CM Sarwer DB Noll JG Wheeler DC Henry KA. The intersection of adverse childhood experiences and neighborhood determinants of health: An exploratory spatial analysis. psychol Trauma: Theory Res Pract Policy. (2024) 16:S125–32. doi: 10.1037/tra0001320 PMC9839886 35834220

[4] Campbell JA Walker RJ Egede LE. Associations between adverse childhood experiences, high-risk behaviors, and morbidity in adulthood. Am J Prev Med. (2016) 50:344–52. doi: 10.1016/j.amepre.2015.07.022 PMC4762720 26474668

[5] Merrick MT Ford DC Ports KA Guinn AS Chen J Klevens J. Vital signs: estimated proportion of adult health problems attributable to adverse childhood experiences and implications for prevention — 25 states, 2015–2017. Morb Mortal Wkly Rep. (2019) 68:999–1005. doi: 10.15585/mmwr.mm6844e1 PMC6837472 31697656

[6] Felitti VJ Anda RF Nordenberg D Williamson DF Spitz AM Edwards V. Relationship of childhood abuse and household dysfunction to many of the leading causes of death in adults: the adverse childhood experiences (ACE) study. Am J Prev Med. (2019) 56:774–86. doi: 10.1016/j.amepre.2019.04.001 31104722

[7] Forrest LN Grilo CM Udo T. Suicide attempts among people with eating disorders and adverse childhood experiences: Results from a nationally representative sample of adults. Int J Eat Disord. (2021) 54:326–35. doi: 10.1002/eat.23457 33372308

[8] Grigsby TJ Rogers CJ Albers LD Benjamin SM Lust K Eisenberg ME. Adverse childhood experiences and health indicators in a young adult, college student sample: differences by gender. Int J Behav Med. (2020) 27:660–7. doi: 10.1007/s12529-020-09913-5 32643038

[9] Peterson C Aslam MV Niolon PH Bacon S Bellis MA Mercy JA. Economic burden of health conditions associated with adverse childhood experiences among US adults. JAMA Netw Open. (2023) 6:e2346323. doi: 10.1001/jamanetworkopen.2023.46323 38055277 PMC10701608

[10] Wiss DA Brewerton TD. Adverse childhood experiences and adult obesity: A systematic review of plausible mechanisms and meta-analysis of cross-sectional studies. Physiol Behav. (2020) 223:112964. doi: 10.1016/j.physbeh.2020.112964 32479804

[11] Aquilina SR Shrubsole MJ Butt J Sanderson M Schlundt DG Cook MC. Adverse childhood experiences and adult diet quality. J Nutr Sci. (2021) 10:e95. doi: 10.1017/jns.2021.85 34804516 PMC8596075

[12] de los Angeles WWL. Association between adverse childhood experiences and diet, exercise, and sleep in pre-adolescents. Acad Pediatr. (2022) 22:1281–6. doi: 10.1016/j.acap.2022.06.007 35728730

[13] Windle M Haardörfer R Getachew B Shah J Payne J Pillai D. A multivariate analysis of adverse childhood experiences and health behaviors and outcomes among college students. J Am Coll Heal. (2018) 66:246–51. doi: 10.1080/07448481.2018.1431892 PMC5948167 29405856

[14] Monteiro CA Cannon G Moubarac JC Levy RB Louzada MLC Jaime PC. The UN Decade of Nutrition, the NOVA food classification and the trouble with ultra-processing. Public Heal Nutr. (2018) 21:5–17. doi: 10.1017/s1368980017000234 PMC10261019 28322183

[15] Gearhardt AN Corbin WR Brownell KD. Development of the yale food addiction scale version 2. 0 Psychol Addict Behav. (2016) 30:113–21. doi: 10.1037/adb0000136 26866783

[16] American Psychiatric Association. Feeding and eating disorders. In: Diagnostic and Statistical Manual of Mental Disorders (5th ed). American Psychiatric Publishing. doi: 10.1176/appi.books.9780890425787.x10_Feeding_and_Eating_Disorders

[17] Wiss DA Brewerton TD Tomiyama AJ. Limitations of the protective measure theory in explaining the role of childhood sexual abuse in eating disorders, addictions, and obesity: an updated model with emphasis on biological embedding. Eat Weight Disord Stud Anorex Bulim Obes. (2022) 27:1249–67. doi: 10.1007/s40519-021-01293-3 34476763

[18] Lund JI Toombs E Radford A Boles K Mushquash C. Adverse childhood experiences and executive function difficulties in children: A systematic review. Child Abus Negl. (2020) 106:104485. doi: 10.1016/j.chiabu.2020.104485 32388225

[19] Dempster KS O’Leary DD MacNeil AJ Hodges GJ Wade TJ. Linking the hemodynamic consequences of adverse childhood experiences to an altered HPA axis and acute stress response. Brain Behav Immun. (2021) 93:254–63. doi: 10.1016/j.bbi.2020.12.018 33358983

[20] Voellmin A Winzeler K Hug E Wilhelm FH Schaefer V Gaab J. Blunted endocrine and cardiovascular reactivity in young healthy women reporting a history of childhood adversity. Psychoneuroendocrinology. (2015) 51:58–67. doi: 10.1016/j.psyneuen.2014.09.008 25290347

[21] Dillon DG Holmes AJ Birk JL Brooks N Lyons-Ruth K Pizzagalli DA. Childhood adversity is associated with left basal ganglia dysfunction during reward anticipation in adulthood. Biol Psychiatry. (2009) 66:206–13. doi: 10.1016/j.biopsych.2009.02.019 PMC2883459 19358974

[22] Anda RF Felitti VJ Bremner JD Walker JD Whitfield CH Perry BD. The enduring effects of abuse and related adverse experiences in childhood. Eur Arch Psychiatry Clin Neurosci. (2006) 256:174–86. doi: 10.1007/s00406-005-0624-4 PMC3232061 16311898

[23] Oswald LM Wand GS Kuwabara H Wong DF Zhu S Brasic JR. History of childhood adversity is positively associated with ventral striatal dopamine responses to amphetamine. Psychopharmacology. (2014) 231:2417–33. doi: 10.1007/s00213-013-3407-z PMC4040334 24448898

[24] Grusnick JM Garacci E Eiler C Williams JS Egede LE. The association between adverse childhood experiences and personality, emotions and affect: Does number and type of experiences matter? J Res Pers. (2020) 85:103908. doi: 10.1016/j.jrp.2019.103908 32863469 PMC7453784

[25] Boyes ME Hasking PA Martin G. Adverse life experience and psychological distress in adolescence: moderating and mediating effects of emotion regulation and rumination. Stress Heal. (2016) 32:402–10. doi: 10.1002/smi.2635 25764473

[26] Cummings JR Schiestl ET Tomiyama AJ Mamtora T Gearhardt AN. Highly processed food intake and immediate and future emotions in everyday life. Appetite. (2022) 169:105868. doi: 10.1016/j.appet.2021.105868 34915102 PMC8886797

[27] Vidal EJ Alvarez D Martinez-Velarde D Vidal-Damas L Yuncar-Rojas KA Julca-Malca A. Perceived stress and high fat intake: A study in a sample of undergraduate students. PloS One. (2018) 13:e0192827. doi: 10.1371/journal.pone.0192827 29522535 PMC5844534

[28] van Strien T Gibson EL Baños R Cebolla A Winkens LHH. Is comfort food actually comforting for emotional eaters? A (moderated) mediation analysis. Physiol Behav. (2019) 211:112671. doi: 10.1016/j.physbeh.2019.112671 31484047

[29] Bui C Lin LY Wu CY Chiu YW Chiou HY. Association between emotional eating and frequency of unhealthy food consumption among Taiwanese adolescents. Nutrients. (2021) 13:2739. doi: 10.3390/nu13082739 34444899 PMC8401002

[30] Hemmingsson E. Early childhood obesity risk factors: socioeconomic adversity, family dysfunction, offspring distress, and junk food self-medication. Curr Obes Rep. (2018) 7:204–9. doi: 10.1007/s13679-018-0310-2 PMC5958160 29704182

[31] Testa A Zhang L Jackson DB Ganson KT Raney JH Nagata JM. Adverse childhood experiences and unhealthy dietary behaviours in adulthood. Public Heal Nutr. (2024) 27:e40. doi: 10.1017/s1368980024000144 PMC10882537 38234114

[32] Wiss DA LaFata EM. Structural equation modeling of adverse childhood experiences, ultra-processed food intake, and symptoms of post-traumatic stress disorder, ultra-processed food addiction, and eating disorder among adults seeking nutrition counseling in Los Angeles, CA. Appetite. (2025) 208:107938. doi: 10.1016/j.appet.2025.107938 40031408

[33] Moubarac JC Batal M Louzada ML Steele EM Monteiro CA. Consumption of ultra-processed foods predicts diet quality in Canada. Appetite. (2017) 108:512–20. doi: 10.1016/j.appet.2016.11.006 27825941

[34] Fernández MS Pilatti A Pautassi RM. Eating-to-cope motives and uncontrolled eating as mediators between negative emotional states and food addiction among Argentinean young adults. Int J Ment Heal Addict. (2024) 22:1433–51. doi: 10.1007/s11469-022-00934-7 PMC9579650 36275610

[35] Bjorlie K Forbush KT Chapa DAN Richson BN Johnson SN Fazzino TL. Hyper-palatable food consumption during binge-eating episodes: A comparison of intake during binge eating and restricting. Int J Eat Disord. (2022) 55:688–96. doi: 10.1002/eat.23692 35194821

[36] Ayton A Ibrahim A Dugan J Galvin E Wright OW. Ultra-processed foods and binge eating: A retrospective observational study. Nutrition. (2021) 84:111023. doi: 10.1016/j.nut.2020.111023 33153827

[37] Ayton A Ibrahim A. The Western diet: a blind spot of eating disorder research?—a narrative review and recommendations for treatment and research. Nutr Rev. (2020) 78:579–96. doi: 10.1093/nutrit/nuz089 PMC7682725 31846028

[38] Takgbajouah M Barnes N MacKillop J Murphy JG Buscemi J. The role of anhedonia in the relationship between adverse childhood experiences (ACEs), alcohol use disorder symptoms, and food addiction symptoms in a sample of emerging adults with histories of heavy drinking. Exp Clin Psychopharmacol. (2023) 32:418–27. doi: 10.1037/pha0000703 PMC11984557 38127517

[39] Lim MSM Cheung FYL Kho JM Tang CSK. Childhood adversity and behavioural addictions: the mediating role of emotion dysregulation and depression in an adult community sample. Addict Res Theory. (2020) 28:116–23. doi: 10.1080/16066359.2019.1594203

[40] Ghaffari A Graves KY Hogans-Mathews S Flowers K Harman JS. Associations of adverse childhood events with disordered eating behaviors among US adolescents. Eat Behav. (2024) 55:101929. doi: 10.1016/j.eatbeh.2024.101929 39447402

[41] Hazzard VM Yoon C Emery RL Mason SM Crosby RD Wonderlich SA. Adverse childhood experiences in relation to mood-, weight-, and eating-related outcomes in emerging adulthood: Does self-compassion play a buffering role? Child Abus Negl. (2021) 122:105307. doi: 10.1016/j.chiabu.2021.105307 PMC8612957 34492573

[42] Horvath S Cox S Tabone J Tabone L Szoka N Abunnaja S. Binge eating in patients pursuing bariatric surgery: understanding relationships with food insecurity and adverse childhood experiences. Surg Obes Relat Dis. (2023) 19:484–90. doi: 10.1016/j.soard.2022.11.003 36528545

[43] Yoon CY Mason SM Loth K Jacobs DR. Adverse childhood experiences and disordered eating among middle-aged adults: Findings from the coronary artery risk development in young adults study. Prev Med. (2022) 162:107124. doi: 10.1016/j.ypmed.2022.107124 35787840 PMC12755113

[44] Nelson JD Martin LN Izquierdo A Kornienko O Cuellar AE Cheskin LJ. The role of discrimination and adverse childhood experiences in disordered eating. J Eat Disord. (2023) 11:29. doi: 10.1186/s40337-023-00753-8 36850009 PMC9969653

[45] Kovács-Tóth B Oláh B Szabó IK Túry F. Adverse childhood experiences increase the risk for eating disorders among adolescents. Front Psychol. (2022) 13:1063693. doi: 10.3389/fpsyg.2022.1063693 36578685 PMC9791097

[46] Chu J Raney JH Ganson KT Wu K Rupanagunta A Testa A. Adverse childhood experiences and binge-eating disorder in early adolescents. J Eat Disord. (2022) 10:168. doi: 10.1186/s40337-022-00682-y 36384578 PMC9670461

[47] Rienecke RD Johnson C Grange DL Manwaring J Mehler PS Duffy A. Adverse childhood experiences among adults with eating disorders: comparison to a nationally representative sample and identification of trauma profiles. J Eat Disord. (2022) 10:72. doi: 10.1186/s40337-022-00594-x 35596196 PMC9123748

[48] Rienecke RD Johnson C Mehler PS Grange DL Manwaring J Duffy A. Adverse childhood experiences among a treatment-seeking sample of adults with eating disorders. Eur Eat Disord Rev. (2022) 30:156–67. doi: 10.1002/erv.2880 35001471

[49] Gomez B Peh CX Cheok C Guo S. Adverse childhood experiences and illicit drug use in adolescents: Findings from a national addictions treatment population in Singapore. J Subst. (2018) 23:86–91. doi: 10.1080/14659891.2017.1348558

[50] Moss HB Ge S Trager E Saavedra M Yau M Ijeaku I. Risk for Substance Use Disorders in young adulthood: Associations with developmental experiences of homelessness, foster care, and adverse childhood experiences. Compr Psychiatry. (2020) 100:152175. doi: 10.1016/j.comppsych.2020.152175 32345436

[51] Hoover LV Yu HP Cummings JR Ferguson SG Gearhardt AN. Co-occurrence of food addiction, obesity, problematic substance use, and parental history of problematic alcohol use. Psychol Addict Behav. (2022) 37:928–35. doi: 10.1037/adb0000870 PMC10986778 35878078

[52] Gearhardt AN Davis C Kuschner R Brownell KD. The addiction potential of hyperpalatable foods. Curr Drug Abus Rev. (2011) 4:140–5. doi: 10.2174/1874473711104030140 21999688

[53] Volkow ND Wise RA Baler R. The dopamine motive system: implications for drug and food addiction. Nat Rev Neurosci. (2017) 18:741–52. doi: 10.1038/nrn.2017.130 29142296

[54] Garfield JBB Lubman DI Yücel M. Anhedonia in substance use disorders: A systematic review of its nature, course and clinical correlates. Aust N Z J Psychiatry. (2014) 48:36–51. doi: 10.1177/0004867413508455 24270310

[55] Gearhardt AN Yokum S Orr PT Stice E Corbin WR Brownell KD. Neural correlates of food addiction. Arch Gen Psychiatry. (2011) 68:808–16. doi: 10.1001/archgenpsychiatry.2011.32 PMC3980851 21464344

[56] Burger KS. Frontostriatal and behavioral adaptations to daily sugar-sweetened beverage intake: a randomized controlled trial 1–3. Am J Clin Nutr. (2017) 105:555–63. doi: 10.3945/ajcn.116.140145 PMC5320411 28179221

[57] Mies GW Treur JL Larsen JK Halberstadt J Pasman JA Vink JM. The prevalence of food addiction in a large sample of adolescents and its association with addictive substances. Appetite. (2017) 118:97–105. doi: 10.1016/j.appet.2017.08.002 28826746

[58] Nordin ASA Adamson SJ Sellman JD. Food addiction does not explain weight gain in smoking cessation. J Smok Cessat. (2018) 13:59–62. doi: 10.1017/jsc.2017.4

[59] Koball AM Glodosky NC Ramirez LD Kallies KJ Gearhardt AN. From substances to food: an examination of addiction shift in individuals undergoing residential treatment for substance use. Addict Res Theory. (2019) 27:322–7. doi: 10.1080/16066359.2018.1516757

[60] Casement MD Shaw DS Sitnick SL Musselman SC Forbes EE. Life stress in adolescence predicts early adult reward-related brain function and alcohol dependence. Soc Cognit Affect Neurosci. (2015) 10:416–23. doi: 10.1093/scan/nsu061 PMC4350480 24795442

[61] Wardell JD Strang NM Hendershot CS. Negative urgency mediates the relationship between childhood maltreatment and problems with alcohol and cannabis in late adolescence. Addict Behav. (2016) 56:1–7. doi: 10.1016/j.addbeh.2016.01.003 26774820 PMC5266596

[62] Gerhardt S Eidenmueller K Hoffmann S Bekier NK Bach P Hermann D. A history of childhood maltreatment has substance- and sex-specific effects on craving during treatment for substance use disorders. Front Psychiatry. (2022) 13:866019. doi: 10.3389/fpsyt.2022.866019 35492729 PMC9046680

[63] Elton A Smitherman S Young J Kilts CD. Effects of childhood maltreatment on the neural correlates of stress- and drug cue-induced cocaine craving. Addict Biol. (2015) 20:820–31. doi: 10.1111/adb.12162 PMC4362751 25214317

[64] Osadchiy V Mayer EA Bhatt R Labus JS Gao L Kilpatrick LA. History of early life adversity is associated with increased food addiction and sex-specific alterations in reward network connectivity in obesity. Obes Sci Pr. (2019) 5:416–36. doi: 10.1002/osp4.362 PMC6819979 31687167

[65] Hoover LV Yu HP Duval ER Gearhardt AN. Childhood trauma and food addiction: The role of emotion regulation difficulties and gender differences. Appetite. (2022) 177:106137. doi: 10.1016/j.appet.2022.106137 35738482

[66] Sharma S Fernandes MF Fulton S. Adaptations in brain reward circuitry underlie palatable food cravings and anxiety induced by high-fat diet withdrawal. Int J Obes. (2013) 37:1183–91. doi: 10.1038/ijo.2012.197 23229740

[67] Lowe MR. The effects of dieting on eating behavior: A three-factor model. Psychol Bull. (1993) 114:100–21. doi: 10.1037/0033-2909.114.1.100 8346324

[68] Stice E Rohde P Shaw H Desjardins C. Weight suppression increases odds for future onset of anorexia nervosa, bulimia nervosa, and purging disorder, but not binge eating disorder. Am J Clin Nutr. (2020) 112:941–7. doi: 10.1093/ajcn/nqaa146 PMC7528557 32534455

[69] Keel PK Heatherton TF. Weight suppression predicts maintenance and onset of bulimic syndromes at 10-year follow-up. J Abnorm Psychol. (2010) 119:268–75. doi: 10.1037/a0019190 PMC2869470 20455599

[70] BodellLPRacineSEWildesJE. Examining weight suppression as a predictor of eating disorder symptom trajectories in anorexia nervosa. Int J Eat Disord. (2016) 49:753–63. doi: 10.1002/eat.22545 27084065

[71] Hagan KE Clark KE Forbush KT. Incremental validity of weight suppression in predicting clinical impairment in bulimic syndromes. Int J Eat Disord. (2017) 50:672–8. doi: 10.1002/eat.22673 28093836

[72] French SA Jeffery RW. Current dieting, weight loss history, and weight suppression: Behavioral correlates of three dimensions of dieting. Addict Behav. (1997) 22:31–44. doi: 10.1016/s0306-4603(96)00002-0 9022870

[73] Stice E Durant S Burger KS Schoeller DA. Weight suppression and risk of future increases in body mass: effects of suppressed resting metabolic rate and energy expenditure. Am J Clin Nutr. (2011) 94:7–11. doi: 10.3945/ajcn.110.010025 21525201 PMC3127521

[74] Lowe MR Marti CN Lesser EL Stice E. Weight suppression uniquely predicts body fat gain in first-year female college students. Eat Behav. (2019) 32:60–4. doi: 10.1016/j.eatbeh.2018.11.005 30594109

[75] Keel PK Bodell LP Haedt-Matt AA Williams DL Appelbaum J. Weight suppression and bulimic syndrome maintenance: Preliminary findings for the mediating role of leptin. Int J Eat Disord. (2017) 50:1432–6. doi: 10.1002/eat.22788 PMC5752142 29044587

[76] Bodell LP Keel PK. Weight suppression in bulimia nervosa: associations with biology and behavior. J Abnorm Psychol. (2015) 124:994–1002. doi: 10.1037/abn0000077 26191637 PMC4658277

[77] Lowe MR Singh S Rosenbaum M Mayer L. Physiological, body composition, and body mass measures show that a developmental measure of weight suppression is more valid than the traditional measure. Int J Eat Disord. (2024) 57:1599–608. doi: 10.1002/eat.24210 PMC11949195 38597163

[78] Lowe MR Piers AD Benson L. Weight suppression in eating disorders: a research and conceptual update. Curr Psychiatry Rep. (2018) 20:80. doi: 10.1007/s11920-018-0955-2 30155651

[79] Goodman EL Baker JH Peat CM Yilmaz Z Bulik CM Watson HJ. Weight suppression and weight elevation are associated with eating disorder symptomatology in women age 50 and older: Results of the gender and body image study. Int J Eat Disord. (2018) 51:835–41. doi: 10.1002/eat.22869 PMC6381935 29693735

[80] Lavender JM Shaw JA Crosby RD Feig EH Mitchell JE Crow SJ. Associations between weight suppression and dimensions of eating disorder psychopathology in a multisite sample. J Psychiatr Res. (2015) 69:87–93. doi: 10.1016/j.jpsychires.2015.07.021 26343599 PMC4561862

[81] Romano KA Heron KE Ebener D. Associations among weight suppression, self-acceptance, negative body image, and eating disorder behaviors among women with eating disorder symptoms. Women Heal. (2021) 61:791–9. doi: 10.1080/03630242.2021.1970082 PMC8440428 34433381

[82] Bodell LP Brown TA Keel PK. Weight suppression predicts bulimic symptoms at 20-year follow-up: the mediating role of drive for thinness. J Abnorm Psychol. (2017) 126:32–7. doi: 10.1037/abn0000217 PMC5215971 27808544

[83] Smith CE Sinclair KL Erinosho T Pickett AC Kercher VMM Ciciolla L. Associations between adverse childhood experiences and history of weight cycling. Obes Sci Pr. (2024) 10:e736. doi: 10.1002/osp4.736 PMC10870800 38371174

[84] Lagan S Shott ME Frank GKW. Adverse childhood experiences, low self-esteem, and salient stimulus response in eating disorders. Eur Eat Disord Rev. (2024) 32:618–32. doi: 10.1002/erv.3064 38349113

[85] Keel PK Bodell LP Forney KJ Appelbaum J Williams D. Examining weight suppression as a transdiagnostic factor influencing illness trajectory in bulimic eating disorders. Physiol Behav. (2019) 208:112565. doi: 10.1016/j.physbeh.2019.112565 31153878 PMC6636832

[86] Keel PK Haedt-Matt AA Hildebrandt B Bodell LP Wolfe BE Jimerson DC. Satiation deficits and binge eating: Probing differences between bulimia nervosa and purging disorder using an ad lib test meal. Appetite. (2018) 127:119–25. doi: 10.1016/j.appet.2018.04.009 PMC5994372 29654850

[87] Wagner A Aizenstein H Venkatraman VK Fudge J May JC Mazurkewicz L. Altered reward processing in women recovered from anorexia nervosa. Am J Psychiatry. (2007) 164:1842–9. doi: 10.1176/appi.ajp.2007.07040575 18056239

[88] Wagner A Aizenstein H Venkatraman VK Bischoff-Grethe A Fudge J May JC. Altered striatal response to reward in bulimia nervosa after recovery. Int J Eat Disord. (2010) 43:289–94. doi: 10.1002/eat.20699 PMC4286149 19434606

[89] Wingenfeld K Schäfer I Terfehr K Grabski H Driessen M Grabe H. Reliable, valide und ökonomische Erfassung früher Traumatisierung: Erste psychometrische Charakterisierung der deutschen Version des Adverse Childhood Experiences Questionnaire (ACE). Psychother Psychosom Med Psychol. (2011) 61:e10–4. doi: 10.1055/s-0030-1263161 20878600

[90] Schulte EM Gearhardt AN. Development of the modified Yale food addiction scale version 2.0. Eur Eat Disord Rev. (2017) 25:302–8. doi: 10.1002/erv.2515 28370722

[91] Carter JC Wijk MV Rowsell M. Symptoms of ‘food addiction’ in binge eating disorder using the Yale Food Addiction Scale version 2. 0 Appetite. (2019) 133:362–9. doi: 10.1016/j.appet.2018.11.032 30508614

[92] Gideon N Hawkes N Mond J Saunders R Tchanturia K Serpell L. Development and psychometric validation of the EDE-QS, a 12 item short form of the eating disorder examination questionnaire (EDE-Q). PloS One. (2016) 11:e0152744. doi: 10.1371/journal.pone.0152744 27138364 PMC4854480

[93] Fairburn CG Beglin SJ. Assessment of eating disorders: Interview or self-report questionnaire? Int J Eating Disord. (1994) 16:363–70. doi: 10.1002/1098-108x(199412)16:4<363::aid-eat2260160405>3.0.co;2- 7866415

[94] Prnjak K Mitchison D Griffiths S Mond J Gideon N Serpell L. Further development of the 12-item EDE-QS: identifying a cut-off for screening purposes. BMC Psychiatry. (2020) 20:146. doi: 10.1186/s12888-020-02565-5 32245441 PMC7118929

[95] Wiss DA LaFata EM Tomiyama AJ. A novel weight suppression score associates with distinct eating disorder and ultra-processed food addiction symptoms compared to the traditional weight suppression measure among adults seeking outpatient nutrition counseling. J Eat Disord. (2024) 12:75. doi: 10.1186/s40337-024-01029-5 38853271 PMC11162565

[96] Brewerton TD. Food addiction as a proxy for eating disorder and obesity severity, trauma history, PTSD symptoms, and comorbidity. Eat Weight Disord Stud Anorex Bulim Obes. (2017) 22:241–7. doi: 10.1007/s40519-016-0355-8 28361213

[97] Mahboub N Rizk R Karavetian M de Vries N. Nutritional status and eating habits of people who use drugs and/or are undergoing treatment for recovery: a narrative review. Nutr Rev. (2020) 79:627–35. doi: 10.1093/nutrit/nuaa095 PMC8114851 32974658

[98] StataCorp. Stata Statistical Software: Release 18. College Station, TX: StataCorp LLC (2023).

[99] Wiss DA Avena N Gold M. Food addiction and psychosocial adversity: biological embedding, contextual factors, and public health implications. Nutrients. (2020) 12:3521. doi: 10.3390/nu12113521 33207612 PMC7698089

[100] Brunault P Salamé E Jaafari N Courtois R Réveillère C Silvain C. Why do liver transplant patients so often become obese? The addiction transfer hypothesis. Méd Hypotheses. (2015) 85:68–75. doi: 10.1016/j.mehy.2015.03.026 25896392

[101] Campana B Brasiel PG de Aguiar AS Dutra SCPL. Obesity and food addiction: Similarities to drug addiction. Obes Med. (2019) 16:100136. doi: 10.1016/j.obmed.2019.100136

[102] di Giacomo E Aliberti F Pescatore F Santorelli M Pessina R Placenti V. Disentangling binge eating disorder and food addiction: a systematic review and meta-analysis. Eat Weight Disord Stud Anorex Bulim Obes. (2022) 27:1963–70. doi: 10.1007/s40519-021-01354-7 PMC9287203 35041154

[103] Butryn ML Juarascio A Lowe MR. The relation of weight suppression and BMI to bulimic symptoms. Int J Eat Disord. (2011) 44:612–7. doi: 10.1002/eat.20881 PMC4852137 21997424

[104] Clarke DM McKenzie DP. A caution on the use of cut-points applied to screening instruments or diagnostic criteria. J Psychiatr Res. (1994) 28:185–8. doi: 10.1016/0022-3956(94)90029-9 7932280

[105] Gearhardt AN White MA Masheb RM Morgan PT Crosby RD Grilo CM. An examination of the food addiction construct in obese patients with binge eating disorder. Int J Eat Disord. (2012) 45:657–63. doi: 10.1002/eat.20957 PMC3375872 22684991

[106] Wiss D Brewerton T. Separating the signal from the noise: how psychiatric diagnoses can help discern food addiction from dietary restraint. Nutrients. (2020) 12:2937. doi: 10.3390/nu12102937 32992768 PMC7600542

[107] Wiss DA Waterhous TS. Nutrition therapy for eating disorders, substance use disorders, and addictions. In: Eating Disorders, Addictions and Substance Use Disorders, Research, Clinical and Treatment Perspectives. Brewerton TD Dennis AB, editors. Berlin, Heidelberg: Springer Berlin Heidelberg (2014). p. 509–32. doi: 10.1007/978-3-642-45378-6_23

[108] Wiss DA. The role of nutrition in addiction recovery: what we know and what we don’t. In: The Assessment and Treatment of Addiction. Danovitch I Mooney LJ, editors. Elsevier (2019). p. 21–42. doi: 10.1016/b978-0-323-54856-4.00002-x

