# Supplementary material for: The association between cumulative adverse childhood experiences and ultra-processed food addiction is moderated by substance use disorder history among adults seeking outpatient nutrition counseling
Source: Front Psychiatry. 2025 Mar 27;16:1543923. doi: 10.3389/fpsyt.2025.1543923 (PMC11983559; doi:10.3389/fpsyt.2025.1543923)
Supplement: Supplementary file 1 [file Table1.docx]

| **Supplement A: Logistic Regression Interacting Adverse Childhood Experiences and Substance Use Disorder on Ultra-Processed Food Addiction (N=287; Ages 21+)** | | | |
| --- | --- | --- | --- |
| **UPFA+** | **OR** | **95% CI** | **p-value** |
| **4+ ACEs** | 1.01 | 0.44 - 2.32 | 0.98 |
| **Lifetime SUD** | 1.24 | 0.58 - 2.66 | 0.58 |
| **4+ ACEs#Lifetime SUD** | 2.50 | 0.84 - 7.49 | 0.10 |
| **Age (years)** |  |  |  |
| 18-29 | **-** | **-** | **-** |
| 30-39 | 0.45 | 0.22 - 0.93 | 0.03* |
| 40-49 | 0.98 | 0.42 - 2.32 | 0.97 |
| 50+ | 0.55 | 0.26 - 1.18 | 0.13 |
| **Gender** |  |  |  |
| Not Woman | **-** | **-** | **-** |
| Woman | 1.75 | 0.91 - 3.38 | 0.10 |
| **Race/Ethnicity** |  |  |  |
| Not White | 1.16 | 0.58 - 2.32 | 0.68 |
| White | **-** | **-** | **-** |
| **Education** |  |  |  |
| HS or Less | 0.48 | 0.15 - 1.54 | 0.22 |
| Some College | 0.38 | 0.17 - 0.82 | 0.01* |
| College | 0.64 | 0.33 - 1.24 | 0.19 |
| Graduate School | **-** | **-** | **-** |
| **Parental Education** |  |  |  |
| Not College Grad | 0.67 | 0.37 - 1.21 | 0.18 |
| College Grad | **-** | **-** | **-** |
| **BMI** |  |  |  |
| Underweight | 1.18 | 0.34 - 4.03 | 0.79 |
| Normal Weight | **-** | **-** | **-** |
| Overweight | 2.63 | 1.27 - 5.47 | 0.01** |
| Obesity | 3.10 | 1.59 - 6.02 | 0.00** |
| **Constant** | 0.40 | 0.13 - 1.23 | 0.11 |
| UPFA: Ultra-Processed Food Addiction; OR: Odds Ratio; CI: Confidence Interval | | | |
| ACEs: Adverse Childhood Experiences; SUD: Substance Use Disorder; HS: High School; BMI: Body Mass Index | | | |
| *Significant at p<0.05; **significant at p<0.01 | | |  |
