# Supplementary material for: The association between cumulative adverse childhood experiences and ultra-processed food addiction is moderated by substance use disorder history among adults seeking outpatient nutrition counseling
Source: Front Psychiatry. 2025 Mar 27;16:1543923. doi: 10.3389/fpsyt.2025.1543923 (PMC11983559; doi:10.3389/fpsyt.2025.1543923)
Supplement: Supplementary file 2 [file Table2.docx]

| **Supplement B: Logistic Regression Interacting Adverse Childhood Experiences and Weight Suppression on Eating Disorder (N=287; Ages 21+)** | | | |
| --- | --- | --- | --- |
| **ED+** | **OR** | **95% CI** | **p-value** |
| **4+ ACEs** | 1.09 | 0.51 - 2.29 | 0.83 |
| **WS** | 0.49 | 0.23 - 1.03 | 0.06 |
| **4+ ACEs#WS** | 1.70 | 0.60 - 4.81 | 0.32 |
| **Age (years)** |  |  |  |
| 18-29 | **-** | **-** | **-** |
| 30-39 | 0.75 | 0.36 - 1.57 | 0.45 |
| 40-49 | 0.57 | 0.24 - 1.38 | 0.21 |
| 50+ | 0.68 | 0.32 - 1.48 | 0.34 |
| **Gender** |  |  |  |
| Not Woman | **-** | **-** | **-** |
| Woman | 2.29 | 1.19 - 4.43 | 0.01* |
| **Race/Ethnicity** |  |  |  |
| Not White | 1.55 | 0.75 - 3.21 | 0.23 |
| White | **-** | **-** | **-** |
| **Education** |  |  |  |
| HS or Less | 1.48 | 0.46 - 4.79 | 0.52 |
| Some College | 0.95 | 0.46 - 1.99 | 0.90 |
| College | 1.67 | 0.85 - 3.26 | 0.14 |
| Graduate School | **-** | **-** | **-** |
| **Parental Education** |  |  |  |
| Not College Grad | 0.78 | 0.43 - 1.41 | 0.41 |
| College Grad | **-** | **-** | **-** |
| **BMI** |  |  |  |
| Underweight | 0.82 | 0.25 - 2.66 | 0.74 |
| Normal Weight | **-** | **-** | **-** |
| Overweight | 2.32 | 1.09 - 4.93 | 0.03* |
| Obesity | 2.99 | 1.45 - 6.19 | 0.00** |
| **Constant** | 0.66 | 0.21 - 2.12 | 0.49 |
| ED: Eating Disorder; OR: Odds Ratio; CI: Confidence Interval | | | |
| ACE: Adverse Childhood Experience: WS: Weight Suppressed; HS: High School; BMI: Body Mass Index | | | |
| *Significant at p<0.05; **significant at p<0.01 | | |  |
